# Editorial: Emerging concepts and evidence in novel approaches for spasticity management

**DOI:** 10.3389/fresc.2023.1327346

**Published:** 2023-11-23

**Authors:** Areerat Suputtitada, Merce Avellanet, Paul Winston

**Affiliations:** ^1^ Department of Rehabilitation Medicine, Faculty of Medicine, Chulalongkorn University, and King Chulalongkorn Memorial Hospital, The Thai Red Cross Society, Bangkok, Thailand; ^2^ Excellent Center for Gait and Motion, King Chulalongkorn Memorial Hospital, Bangkok, Thailand; ^3^ Biomedical Engineering, Chulalongkorn University, Bangkok, Thailand; ^4^ Neurorehabilitation Research Unit, Faculty of Medicine, Chulalongkorn University, Bangkok, Thailand; ^5^ Principles and Practice of Clinical Research (PPCR) Program, Harvard T.H. Chan School of Public Health, Harvard Medical School, Boston, MA, United States; ^6^ Canadian Advances in Neuro-Orthopedics for Spasticity Consortium (CANOSC), Kingston, ON, Canada; ^7^ Rehabilitation Department, Hospital N Sra de Meritxell, Escaldes, Andorra; ^8^ Research Group on Health Sciences, Universitat d’Andorra, Saint Julia de Loria, Andorra; ^9^ Department of Physical Medicine and Rehabilitation, University of British Columbia, Vancouver, BC, Canada

**Keywords:** emerging, concepts, evidence, novel, spasticity

**Editorial on the Research Topic** Emerging concepts and evidence in novel approaches for spasticity management

## Introduction

Many neurological diseases commonly cause spasticity. Spasticity may be associated with several important related symptoms, including; muscle tone, weakness, sleep disturbance, neurogenic bladder caused by detrusor spasticity, and pain. Optimization of spasticity treatment must balance the reduction of muscle spasticity and the related symptoms, as well as the recovery of motor function when possible. Facilitation and modulation of neural plasticity through rehabilitative treatment as early interventions with repetitive goal-oriented intensive therapy, non-invasive brain stimulation, and pharmacological agents are the keys to promoting motor recovery (1). Appropriate treatment with botulinum toxin therapy and neurolysis with phenol or alcohol are examples of the most used treatments to create a transient plastic state of the neuromotor system that allows motor re-learning and recovery in subacute and chronic stages. Improved precision of injections, dilutions, dosages, and the accurate evaluation of spasticity are needed. Neural mechanisms of spasticity do not fully explain the motor dysfunction and recovery in patients with spastic disorders (1). Peripheral non-neural mechanisms are also not fully understood. These all necessitate updated evidence in spasticity management.

This research topic sheds light on recent advances in emerging theories and management of adult and pediatric spasticity. The examples are the hyaluronan hypothesis, dry needling, the hydro-dissection effect on spasticity, technology as non-invasive brain stimulation, peripheral magnetic or electrical stimulation, clinical and instrumental evaluations in spasticity, the precisions of injection, ergonomics during injection, dosage and dilution of botulinum toxin, phenol and alcohol, hyaluronidase, cryoneurolysis, surgical interventions, orthoses, and assistive devices, and even practical guidance for outpatient spasticity management during the coronavirus (covid-19) pandemic.

Numerous breakthroughs and novel theories have emerged in the field of spasticity management; nevertheless, the absence of robust empirical data persists. Hence, the objective of this research topic is to present a comprehensive analysis of recent advancements and offer insights into the existing body of evidence relating to these advancements.

## Highlights of the 6 articles published in this research topic and editorial perspectives

A review (Hashemi et al.): The presence of spasticity in individuals with upper motor neuron problems often leads to severe joint abnormalities, which often require neuro-orthopedic surgical interventions. This study addresses a significant gap in the literature by doing a comprehensive evaluation of the effectiveness of therapies for spasticity in adult patients. A comprehensive search yielded a total of 80 pertinent researches, while 40 trial articles were thoroughly analyzed to identify intervention areas. A lack of randomized controlled trials was observed. Although the absence of standardized evaluation procedures poses a significant difficulty, this review effectively illustrates the indisputable beneficial effects of meticulously chosen surgical interventions on the quality of life for individuals suffering with upper limb spasticity. The necessity for unified methodologies and established protocols becomes crucial to promote evidence-based decision-making in surgical selections and patient referrals.

Editorial perspectives: Access to surgical interventions varies widely by access to expert centres that offer treatment. It may be more accessible in countries that cannot afford botulinum toxins, yet inaccessible in many developed economies. This paper summarized the literature and serves as a review to launch more structured outcome based studies (2). The future quality of evidences should be encourage as high rank as randomized trials without serious limitations, well-performed observational studies with vary large effects (or other qualifying factors), or even moderate rank as randomized trials with serious limitations, well-performed observational studies yielding large effects (3). In addition, the outcome assessment is also need to consensus.

Original research (Li et al.): A thorough examination of a cohort of 68 individuals who were referred for inpatient rehabilitation within a six-month period following their initial occurrence of stroke. According to the findings of the study, the patients, on average, underwent their initial phenol neurolysis for roughly 16.3 weeks, with the earliest being 19 days, and more than half of the patients for within 12 weeks of the occurrence of the stroke. The nerves most frequently targeted were the motor branches of the tibial and sciatic nerves. The early phenol neurolysis resulted in a significant reduction in the total amount of botulinum toxin (BoNT) required for the treatment of spasticity without any observed adverse effects.

Editorial perspectives: There are very few studies that have examined the outcomes of phenol neurolysis (4). There are even fewer prospective studies. This retrospective analysis highlights the need for prospective studies to further assess the contribution of phenol to spasticity care. The future research should explore the risk and benefit, short- term and long-term of phenol neurolysis. Additionally, the alcohol neurolysis (5) and the cryoneurolysis (6) are evidenced. The future quality of evidence should be encouraged as high rank as randomized trials without serious limitations, well-performed observational studies with vary large effects (or other qualifying factors), should be done for all these neurolysis.

A case report (Scobie)**:** An illustration of the utilization of cryoneurotomy for controlling shoulder issues associated with spasticity in a 15-year-old male diagnosed with quadriplegic cerebral palsy, classified as Gross Motor Function Classification System 5 (GMFCS 5) is reported. The patient's lower limb spasticity was managed with botulinum toxin A (BoNT-A) injections. However, the decision was made to exclude the shoulder region from treatment due to concerns regarding the potential spread of the toxin and the risk of aspiration. After performing diagnostic nerve blocks, the patient underwent bilateral cryoneurotomies of the right and left lateral pectoral nerves (LPNs), with a duration of 3.5 min for each lesion. The intervention yielded a substantial enhancement in the range of motion (ROM) of the shoulder, as evidenced by the measurements increasing from 86° to 133° on the right side and from 90° to 139° on the left side. The beneficial effects were maintained during the 9-month follow-up period. Furthermore, the caregiver documented significant improvements in the patient's daily functioning, encompassing tasks such as dressing, bathing, transferring, and the ability to maintain a seated position for extended periods of time.

Editorial perspectives: This case report is the first published study on a rapidly evolving novel usage for a decades old treatment, cryoneurotomy or cryoneurolysis. The longstanding results suggest that longer and larger studies are needed as the literature is growing quickly in this field. Considering the potential benefits associated with cryoneurolysis (6), it is imperative to prioritize the generation of high-quality evidence in future research endeavors. This entails conducting randomized trials without significant restrictions, as well as well-executed observational studies that demonstrate substantial impacts or possess other qualifying features, in order to further investigate the efficacy of cryoneurolysis.

Original research (Shackleton et al.)**:** The study had 16 individuals diagnosed with chronic motor incomplete tetraplegia who participated in a 24-week intervention. The intervention consisted of 60-minute sessions conducted three times per week. Participants were randomly assigned to one of two groups: the robotic locomotor training (RLT) group, which utilized the Ekso GT exoskeleton, or the activity-based therapy (ABT) group, which engaged in a combination of resistance, aerobic, and weight-bearing exercises. Remarkably, both interventions failed to produce a meaningful reduction in spasticity. The level of pain intensity showed an increase in both groups; however, it is worth noting that participants in both therapies saw a significant improvement in their subjective quality of life. The observed incongruity between heightened pain levels and enhanced quality of life underscores the necessity for more investigation via more extensive randomized controlled trials.

Editorial perspectives: This study focuses on the effects of physical activity on spasticity, showing no statistical changes after 24 weeks of neither robotic locomotor training nor activity-based therapy. To highlight, all except one of the participants were male with chronic tetraplegia (2–26 years of injury) AIS C and D. Participants previous physical activity is not specified. Despite the results, dosage and timing of exercise interventions should be considered to analyze the effects in spasticity. Given the significance of incorporating different forms of physical activity or exercises into rehabilitation protocols, it is imperative to promote the advancement of future research in order to enhance the quality of evidence. This entails prioritizing rigorous methodologies and optimizing the intensity, frequency, and duration of each exercise to effectively showcase their respective benefits. Furthermore, it is imperative that individuals with chronic incomplete tetraplegia acquire the necessary capabilities to engage in active exercise regimens. Episodes of pain, spasticity, and various forms of discomfort provide significant barriers to engaging in active exercise. All of these problems have to be effectively managed, prevented, or reduced.

Systematic review and meta-analysis (Massey et al.)**:** The efficacy of non-invasive electrical stimulation (ES) in reducing spasticity in patients with spinal cord injury (SCI) was evaluated. A total of twenty-nine studies were examined, utilizing various assessment tools, including the Ashworth scale, modified Ashworth scale, pendulum test, and Penn spasm frequency scale. Several methods of non-invasive electrostimulation (ES) techniques were analysed,, such as transcutaneous electrical nerve stimulation (TENS), transcutaneous spinal cord stimulation (TSCS), and functional electrical stimulation (FES). The findings from meta-analyses demonstrate a statistically significant decrease in spasticity as measured by the Modified Ashworth Scale (MAS) in randomized controlled trials (RCTs). Analysis of non-RCT studies show an improvement with intervention at short term. The results of the pendulum test did not reveal any statistically significant differences. The neurophysiological outcomes exhibited a deficiency, underscoring the significance of their inclusion in forthcoming investigations. The effectiveness of non-invasive electrical stimulation (ES) relies heavily on the activation of afferent fibers.

Editorial perspectives.: When utilizing the Modified Ashworth Scale (MAS) scores, it has been observed that non-invasive electrical stimulation (ES) techniques exhibit efficacy in enhancing spasticity outcomes subsequent to Spinal Cord Injury (SCI), as compared to a control group. There is no observed correlation between neurophysiologic outcomes and clinical outcomes. The inclusion of papers in this analysis raises concerns about the quality of research, particularly with regards to blinding and a notable proportion of non-randomized trials. Moreover, there is considerable variation in the ES methods employed across various research.

Perspective (Suputtitada): Spasticity, which frequently arises as a result of stroke and upper motor neuron disease, exhibits intricate origins that encompass many sensitization mechanisms. Novel therapeutic approaches, including Extracorporeal Shockwave Therapy (ESWT), repetitive peripheral magnetic stimulation (rPMS), and needling procedures, have been developed to specifically address sensitization mechanisms, thereby presenting promising possibilities for the management of spasticity. ESWT has been suggested to potentially exert its effects via inducing nitric oxide synthesis, altering rheological properties, and disrupting neuromuscular transmission. The administration of rPMS has been found to augment sensory input, activate the cortical regions of the brain, and induce softening of the underlying tissues. The primary objective of needling, particularly when employing sterile water injection, is to mitigate peripheral sensitivity and decrease pain associated with spasticity. Although showing potential, the heterogeneous characteristics of spasticity and variations in research methodologies underscore the necessity for additional investigation of these therapies, with the potential to enhance the quality of life for persons who experience spasticity following a stroke or other neurological disorders.

Editorial perspectives: The expert perspective provides a comprehensive examination of the available evidence relevant to the utilization of innovative methods in the treatment of spasticity. The justification for therapeutic alternatives is derived from the novel understandings about spasticity. It is important to highlight that spasticity encompasses not just a cause-effect relationship, but also several mechanisms that disrupt diverse structures, including sensitization and maladaptive plasticity.

### Clinical implications

Emphasizing the critical importance of rigorously conducted randomized controlled trials (RCTs) or carefully planned observational studies is essential. Nevertheless, within the context of the current interim phase, we recommend the implementation of these innovative techniques in accordance with the relevant clinical practice guidelines, even in the absence of definitive randomized controlled trials. It is imperative to gather comprehensive data on various aspects such as efficacy, side effects, cost effectiveness, and other pertinent factors through cohort studies. Furthermore, the decision to discontinue these procedures if there is little clinical efficacy or unacceptable side effects, and further for the alternating high-evidence treatment, holds significant importance in the context of clinical practice.

## Conclusion

An increasing amount of evidence suggests the effectiveness of various new interventions in the management of spasticity. The treatments encompass extracorporeal shockwave therapy (ESWT), repetitive peripheral magnetic stimulation (rPMS), needling, as well as the potential utilization of mechanical needling and sterile water injection, cryoneurolysis, non-invasive electrical stimulation (ES), robotic locomotor training, and innovative applications of established interventions such as surgical procedures, phenol neurolysis, and physical activity or exercises. This highlights the importance of doing a comprehensive examination of the pathophysiology of spasticity. The identification of these novel findings will contribute to the progress of alternative methodologies for the management of spasticity. The promotion of future evidence quality should give priority to randomized trials that have minimal limitations and well-executed observational studies that demonstrate substantial effects or demonstrate other qualifying factors. This approach ensures that the likelihood of future research significantly altering our level of confidence in the estimated effect is minimal.

## References

[1] Li S Francisco GE Rymer WZ. A new definition of poststroke spasticity and the interference of spasticity with motor recovery from acute to chronic stages. Neurorehabil Neural Repair. (2021) 35(7):601–10. 10.1177/15459683211011214 33978513

[2] Jarratt Barnham I Alahmadi S Spillane B Pick A Lamyman M. Surgical interventions in adult upper limb spasticity management: a systematic review. Hand Surg Rehabil. (2022) 41(4):426–34. 10.1016/j.hansur.2022.04.006 35490985

[3] Brozek JL Akl EA Alonso-Coello P Lang D Jaeschke R Williams JW Grading quality of evidence and strength of recommendations in clinical practice guidelines. Part 1 of 3. An overview of the GRADE approach and grading quality of evidence about interventions. Allergy. (2009) 64(5):669–77. 10.1111/j.1398-9995.2009.01973.x 19210357

[4] Karri J Zhang B Li S. Phenol neurolysis for management of focal spasticity in the distal upper extremity. PM R. (2020) 12(3):246–50. 10.1002/pmrj.12217 31278847

[5] Koh M Leng TY. Alcohol motor blocks: case series and a narrative review. Cureus. (2022) 14(1):e21575. 10.7759/cureus.21575 35228934 PMC8867019

[6] Winston P MacRae F Rajapakshe S Morrissey I Boissonnault È Vincent D Analysis of adverse effects of cryoneurolysis for the treatment of spasticity. Am J Phys Med Rehabil. (2023) 102(11):1008–13. 10.1097/PHM.0000000000002267 37104641

